# Establishing a transdiagnostic dimension of early childhood depressive symptoms using the Preschool Feelings Checklist‐Scale

**DOI:** 10.1002/jcv2.70152

**Published:** 2026-10-09

**Authors:** Nicolas L. Camacho, Kirsten E. Gilbert, Rebecca Tillman, Joan L. Luby, Michael S. Gaffrey

**Affiliations:** ^1^ Department of Psychology & Neuroscience Duke University Durham North Carolina USA; ^2^ School of Medicine Washington University St. Louis Missouri USA; ^3^ Children's Wisconsin Milwaukee Wisconsin USA; ^4^ Division of Pediatric Psychology and Developmental Medicine, Department of Pediatrics Medical College of Wisconsin Milwaukee Wisconsin USA

**Keywords:** depression, early childhood, emotion regulation, executive function, psychometrics, reward, transdiagnostic

## Abstract

**Background:**

Young child depressive symptoms are associated with various concurrent and future psychiatric problems, indicating early transdiagnostic risk for psychopathology. We now need studies that identify and test the robustness, reliability, and validity of early emerging depressive symptom dimensions, examining them relative to wide‐ranging forms of psychopathology. Psychometric methods are suitable for such investigations.

**Methods:**

In a sample of 547 young children (ages 3–7 years) enriched for internalizing problems, preregistered analyses on the psychometric properties of the Preschool Feelings Checklist‐Scale (PFC‐S) investigated the robustness and interpretation of young child depressive symptom dimensions using exploratory structural equation models (ESEMs) and correlations alongside related constructs. We also tested the accuracy with which the PFC‐S distinguished children based on broadband psychiatric diagnostic profiles and clinical concern.

**Results:**

A bifactor ESEM with three group factors (Social and Behavioral Anhedonia, Emotional and Behavioral Dysregulation, and Excessive Guilt and Sadness) and residual correlations accounting for item wording similarities was the most valid PFC‐S model (comparative fit index/Tucker‐Lewis index = 0.98; root mean squared error of approximation = 0.06; standardized root mean squared residual = 0.03). Only the general factor emerged as reliable (*ω*_H_ = 0.91; *H* = 0.96; factor determinacy = 0.99). The general factor evidenced correlations with measures of child anhedonia severity, executive dysfunction, and emotion dysregulation (|*r*s| > .60) a well as correlations with child reward responsiveness, driven goal pursuit, inhibited behavior, sex assigned at birth, and caregiver depressive symptom severity (0 < |*r*s| ≤ .37), but was not significantly correlated with child guilt, approach behavior, or fun‐seeking (|*r*s| ≤ .07). The general factor most accurately distinguished children with multiple co‐occurring diagnoses, including depression, and those with no diagnoses, as well as children with versus without clinical levels of general psychopathology.

**Conclusion:**

Findings support a PFC‐S general factor characterized by reward and executive dysfunction with both depression‐specific and transdiagnostic psychopathology risk relevance in young children.

## Key Points

- What's Known?
  ◦ Young child depressive symptoms are predictive of future depression and are associated with various other psychiatric problems.
- What's New?
  ◦ The present investigation examined the psychometric properties of the Preschool Feelings Checklist‐Scale (PFC‐S) in a large sample of young children enriched for internalizing problems.
  ◦ A robust and reliable general factor was identified within a multidimensional model of depressive symptoms, characterized as measuring reward‐related and executive dysfunctions.
  ◦ This general factor emerged as an accurate indicator of diagnostic co‐occurrence, especially alongside depression, and general psychopathology.
- What's Relevant?
  ◦ Findings further support the emerging clinical utility of the PFC‐S as a depression and transdiagnostic psychopathology tool.
  ◦ Findings inform PFC‐S use in studies of early emerging biopsychosocial correlates of reward and executive dysfunctions.

## INTRODUCTION

Major depressive disorder (MDD) can emerge in children during the preschool period (3–5 years old) (Centers for Disease and Control Prevention, 2025), increasing in prevalence from early childhood (6–8 years old) into adolescence (Kieling et al., 2024). Young children (i.e., 3–8 years old) with MDD are at increased risk of exhibiting MDD across childhood and adolescence, demonstrating evidence for homotypic continuity (Gaffrey, Tillman, et al., 2018; Luby et al., 2014; Luby, Si, et al., 2009; Silver et al., 2022). However, MDD also shows heterotypic continuity into adolescence, as depressive symptom elevations in young children are associated with concurrent and prospective risk for the development of anxiety disorders and various disruptive behavior disorders (DBDs), including oppositional defiant disorder (ODD), conduct disorder (CD), and attention‐deficit/hyperactivity disorder (ADHD) (Bufferd et al., 2012; Finsaas et al., 2018; Gaffrey, Tillman, et al., 2018; Luby, Barch, et al., 2018; Luby, Belden, et al., 2009; Luby et al., 2014; Silver et al., 2022). While prior work emphasizes the relevance of young child depressive symptomatology within a broader psychiatric nosology, the dimensions that underlie these associations remain insufficiently characterized.

Prior work supports the possibility that early emerging depressive symptoms reflect transdiagnostic risk for psychopathology. Young child depressive symptoms share common variance with core symptoms of ADHD, anxiety, Obsessive‐Compulsive Disorder, and somatic complaints (Holmes et al., 2021; Olino et al., 2014). Novel dimensional models of psychopathology based on older children and adults, such as the Hierarchical Taxonomy of Psychopathology (Kotov et al., 2017), also highlight that depressive symptoms resemble a heterogeneous set of problems that span cognitive (e.g., attentional challenges), emotional/distress (e.g., irritability, anhedonia), somatoform (e.g., dysregulated sleep), and thought problem (e.g., suicidal ideation) domains (Forbes et al., 2024). However, it is not well understood whether measures of young child depressive symptoms can assess specific symptom dimensions relevant in, but not limited to, depression.

Psychometric assessments of young child depressive symptom measures have the potential to elucidate such dimensions. The dimensionality of young child depressive symptoms has been explicitly investigated in only one prior study. In a large community‐based sample of 3–8‐year‐old children, depressive symptoms measured by the Preschool Feelings Checklist‐Scale (PFC‐S) were reliably reflected by an age‐ and sex‐invariant three‐correlated factor (3‐CF) model that represented construct valid dimensions of social and behavioral anhedonia (SBA), emotional and behavioral dysregulation (EBD), and excessive guilt and sadness (EGS) (Camacho et al., 2025). These findings provide important initial evidence that a multidimensional model of early depressive symptoms may be composed of dimensions that vary in their uniqueness to depression. Yet, it remains unclear whether the PFC‐S also reflects a more general dimension of early emerging depressive symptoms and to what extent each dimension relates to various forms of psychopathology. Moreover, wording similarities among items that load onto the SBA factor were noted in the prior PFC‐S study (Camacho et al., 2025), suggesting that future investigations would benefit from considering such within‐factor characteristics to prevent the overestimation of model parameters and overidentification of factors (Gerbing & Anderson, 1984). As a result, directly testing the extent to which the variance of the SBA factor is explained by trivial item characteristics such as wording similarities would help clarify whether the factor can be interpreted as a substantive measure of anhedonia. To position these identified depressive symptom dimensions as transdiagnostic psychopathology indicators, the multidimensional model of the PFC‐S requires further scrutiny using contemporary psychometric science and best practice guidelines (Clark & Watson, 2019).

Building upon our prior work (Camacho et al., 2025), the present investigation addressed three aims in an independent sample. First, we attempted to conceptually replicate and extend the 3‐CF model of the PFC‐S using a step‐wise bifactor modeling approach. This approach helps clarify the robustness and reliability of group factors by testing the extent to which each factor reflects unique variance unattributable to a general factor and whether group factor scores are substantially explained by the general factor (Reise et al., 2023). Second, we attempted to conceptually replicate and increase the specificity of the construct validity of the reliable PFC‐S factor(s). Third, we tested the extent to which reliable PFC‐S factor(s) were more appropriately indicative of depression specifically versus broader forms of psychopathology using both psychiatric diagnostic data and dimensional broadband psychopathology symptoms. We held the following hypotheses: (1) the PFC‐S data would be best represented by a bifactor model with 3 group factors (3‐bifactor), (2) the SBA factor would represent substantive rather than method variance, (3) reliability estimates would support the use of all resulting factors, (4) the factors would demonstrate conceptually replicated and more specific construct validity, and (5) whereas the general and EBD factors would indicate increased general psychopathology risk not solely limited to depression, the SBA and EGS factors would more narrowly indicate risk for depression and internalizing problems.

## MATERIALS AND METHODS

### Participants

The present investigation involved secondary analyses of baseline data from two studies conducted at Washington University School of Medicine in St. Louis, Missouri. The first was a randomized controlled trial of an adapted version of parent‐child interaction therapy that included an emotion development module to address early‐onset MDD symptoms (PCIT‐ED) (Luby, Barch, et al., 2018). The second is an on‐going study of early emerging heightened performance monitoring and overcontrol (neurodevelopment of overcontrol and anxiety [NOA]). Supporting Information S1: Figure S1 shows the studies' recruitment strategies and inclusion/exclusion criteria. Caregivers provided informed consent and children provided assent. The Duke University Institutional Review Board approved data sharing and secondary analysis (Protocol: 2024‐0484) via a data usage agreement with Washington University School of Medicine (Agreement ID: P24‐07100). Study procedures were conducted in accordance with the Declaration of Helsinki.

Data included 547 children aged 3–7 years and their caregivers (Table 1). This included NOA data on 173 community children and PCIT‐ED data on 292 children recruited to receive early‐onset MDD treatment, 26 healthy comparison participants, and 56 community controls. A large portion of this sample was represented by children who identified as white, whose yearly family income was $100,001+, and/or whose biological mothers reported on their behavior. Child education levels from Daycare to Second Grade were represented.

**TABLE 1:** Sample demographics.

Child characteristics	PCIT‐ED (*n* = 374)		NOA (*n* = 173)		Full sample (*N* = 547)	
	*M* (SD)	Range	*M* (SD)	Range	*M* (SD)	Range
Age (in years)	5.10 (1.04)	3.00–7.42	6.12 (1.13)	4.08–7.92	5.42 (1.17)	3.00–7.92

	*n*	%	*n*	%	*n*	%
Sex assigned at birth						
Female	136	36.36	93	53.76	229	41.86
Male	238	63.64	80	46.24	318	58.14
Race						
Asian	4	1.07	4	2.31	8	1.46
Black	51	13.64	18	10.40	69	12.61
White	280	74.87	127	73.41	407	74.41
Multiracial	39	10.43	22	12.72	61	11.15
Undisclosed	0	0.00	2	1.16	2	0.37
Hispanic	41	10.96	14	8.09	55	10.05
Education						
Daycare	20	5.35	1	0.58	21	3.84
Preschool/kindergarten	230	61.50	94	54.34	324	59.23
First grade	27	7.22	55	31.79	82	14.99
Second grade	0	0.00	18	10.40	18	3.29
Not enrolled/Undisclosed	97	25.94	5	2.89	102	18.65
Yearly family income						
$1–50,000	90	24.06	23	13.3	113	20.66
$50,001–100,000	116	31.02	34	19.66	150	27.41
$100,001+	126	33.69	112	64.74	238	43.51
Undisclosed	42	11.23	4	2.31	46	8.41
Informant						
Biological mother	261	69.79	156	90.17	417	76.23
Biological father	12	3.21	9	5.20	21	3.84
Adoptive mother	12	3.21	3	1.73	15	2.74
Other	6	1.61	2	1.16	8	1.45
Not reported	83	22.19	3	1.73	86	15.72

*Note*: *M* and SD are used to represent mean and standard deviation, respectively.

Abbreviations: NOA, neurodevelopment of overcontrol and anxiety study; PCIT‐ED, parent‐child interaction therapy–emotion development study.

### Measures

The present investigation includes measures of depression‐related constructs and broader transdiagnostic processes (e.g., emotion dysregulation, caregiver depressive severity) previously used in a construct validation of the PFC‐S (Camacho et al., 2025). Moreover, measures of anhedonia, EBD, and guilt were also included to test the specific and transdiagnostic construct(s) hypothesized to be measured by the PFC‐S. Finally, two measures were included to test the PFC‐S as an indicator of diagnostic and dimensional psychiatric problems (i.e., the Kiddie Schedule for Affective Disorders and Schizophrenia–Early Childhood [KSADS‐EC] and Child Behavior Checklist [CBCL]).

#### Preschool Feelings Checklist‐Scale

Caregivers in both studies reported on their children's depressive symptoms over the previous 2 weeks using the PFC‐S. The 23‐item PFC‐S uses a 5‐point scale (0 = *Never*, 1 = *Rarely*, 2 = *Sometimes*, 3 = *Often*, 4 = *Most of the time*) (Supporting Information S1: Table S1; Figure S2) (Camacho et al., 2025; Luby et al., 2020). Item 16 was not included in the analyses of the present investigation, as it has shown poor communality with the other PFC‐S items (Camacho et al., 2025). Higher scores indicate increased depressive symptom severity. The PFC‐S is a candidate measure to study depressive symptom dimensions in young children (Camacho et al., 2025).

#### Emotion Regulation Checklist

The 24‐item caregiver‐rated Emotion Regulation Checklist (ERC) (Shields & Cicchetti, 1997) assesses how children manage their emotions in social settings on a 4‐point scale (1 = *Never* to 4 = *Almost always*). Higher mean scores on the lability/negativity (LN) scale (Cronbach's *α* = .93, average inter‐item correlation [AIC] = 0.49) indicated more labile and extreme expressions of affect, anger, and difficulties transitioning between activities. Higher mean scores on the Emotion Regulation scale (*α* = .85, AIC = 0.41) indicated greater affect modulation and positive responses to friendly overtures.

#### Behavioral Inhibition/Activation Scale

The Behavioral Inhibition/Activation Scale (BIS/BAS) (Carver & White, 1994) is a 20‐item caregiver‐report questionnaire of BIS and BAS systems in children using a 7‐point scale (1 = *Extremely untrue* to 7 = *Extremely true*). Caregivers in the PCIT‐ED study completed this measure. Higher mean BIS scores (*α* = .82, AIC = 0.39) indicated increased sensitivity to potential environmental punishments. Higher mean BAS scores (*α* = .86, AIC = 0.32) indicated increased general approach behaviors that subsume subscales encompassing Reward Responsiveness (*α* = .85, AIC = 0.54), Fun‐Seeking Behavior (*α* = .65, AIC = 0.32), and Drive (in goal pursuit) (*α* = .87, AIC = 0.62).

#### Beck Depression Inventory‐II

The Beck Depression Inventory‐II (BDI‐II) (Beck et al., 1996) is a 21‐item questionnaire that measures the self‐reported depressive symptomatology of caregivers over the previous 2 weeks based on a 4‐point scale (0 = *No more than usual* to 3 = *All the time*). Item 9 (i.e., suicidal thoughts/wishes) was not included in the NOA study because questionnaires were completed outside of the laboratory and it was not feasible for research staff to monitor, provide resources, and/or intervene if elevated suicidal risk was endorsed. Mean scores for both studies were calculated without this item and indicated depressive symptom severity (*α* = .96, AIC = 0.52).

#### Behavior Rating Inventory of Executive Function

The Behavior Rating Inventory of Executive Function (BRIEF) (Gioia et al., 2000) measures child executive functioning as perceived by caregivers. NOA participants 5 years old and younger and PCIT‐ED participants 5.5 years old and younger were evaluated with the 63‐item preschool version (Sherman & Brooks, 2010). All other children were evaluated with the 86‐item school‐age version (Gioia et al., 2000). Caregivers in both studies rated items on a 3‐point scale (0 = *Never*, 1 = *Sometimes*, 2 = *Often*). The BRIEF evaluates domains including inhibition, attention shifting, emotional control, working memory, organization, and planning. Higher *T*‐scores on the global executive composite (GEC) indicate more severe executive dysfunction.

#### My Child 2

The My Child 2 (MC2) (Kochanska et al., 1994) is a caregiver‐report questionnaire of shame, guilt feelings, and associated reparative behaviors in children. Caregivers in the PCIT‐ED study rated 100 items on a 6‐point scale (1 = *Extremely Untrue* to 6 = *Quite True*). Higher mean scores on the Guilt Feelings scale indicated elevated expressions of shame and guilt (*α* = .88, AIC = 0.29) based on the Symbolic Reproduction, Guilt, Concerned by Others' Transgressions, and Sensitive to Themes of Wrongdoing subscales.

#### Kiddie Schedule for Affective Disorders and Schizophrenia–Early Childhood

The KSADS‐EC (Gaffrey & Luby, 2012), an early childhood adaptation of the KSADS (Kaufman & Schweder, 2004) and a semi‐structured clinical interview, was administered to caregivers by master's level researchers in both studies to assess the presence of psychiatric disorders in their children. Community controls in the PCIT‐ED study were not administered the KSADS‐EC and healthy controls were only administered the MDD section. Symptoms were rated on a 3‐point scale (1 = *Not present*, 2 = *Subthreshold*, 3 = *Threshold*). The MDD screener item that assessed the severity of children's recent expressions of loss of pleasure, enjoyment, and interest in preferred activities was used to indicate anhedonia severity.

Children 6 years old and younger were required to exhibit 4+ symptoms (including depressed/irritable mood or loss of interest/pleasure) at least at a Subthreshold level for 1+ week to meet criteria for MDD (Luby et al., 2003). All other diagnostic criteria, including MDD for children 7‐years‐old and older, were unedited. Current diagnoses were used to create 8 groups of young children (Figure 1). Groups were created based on the presence/absence of any diagnosis, whether children had MDD and diagnostic co‐occurrence, or lack thereof. Two groups were created to distinguish between children with MDD or 1+ anxiety disorder, but not both. High co‐occurrence among disorder types, especially involving MDD and DBDs, and low representation of specific diagnoses precluded the formation of additional groups (Supporting Information S1: Figure S3).

**FIGURE 1:**
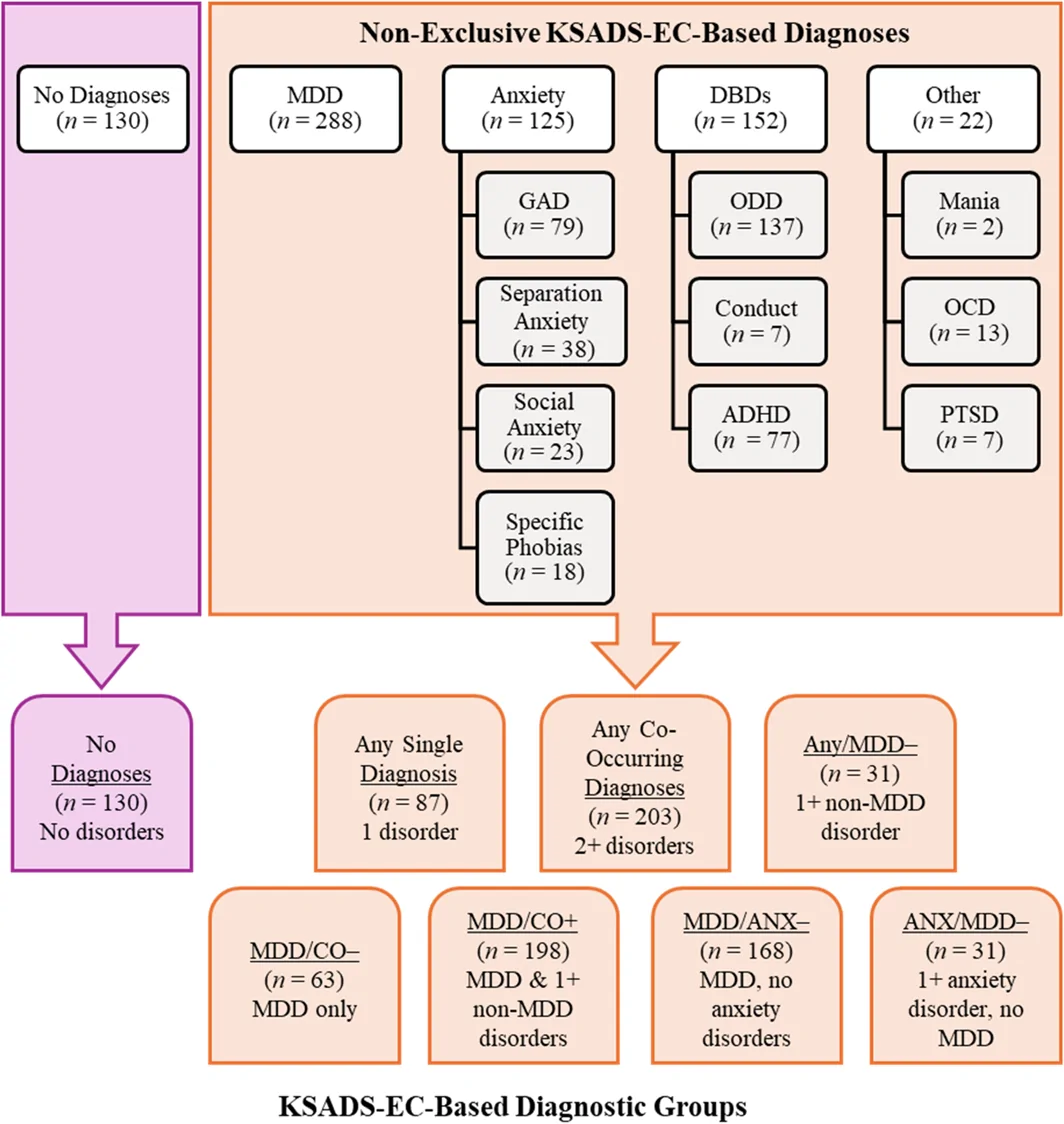
Breakdown of KSADS‐EC‐based diagnoses and diagnostic group specifications. Children with no diagnoses were grouped together. Diagnostic groups were created based on the specified criteria. Children were allowed to have more than 1 diagnosis and were allowed to be included in more than one group. ADHD, attention‐deficit/hyperactivity disorder; ANX, 1+ anxiety diagnosis; ANX−, no anxiety diagnoses; CO−, no co‐occurring diagnoses; CO+, with co‐occurring diagnoses; DBDs, disruptive behavior disorders; GAD, generalized anxiety disorder; KSADS‐EC, Kiddie Schedule for Affective Disorders and Schizophrenia Early Childhood; MDD, major depressive disorder; OCD, obsessive‐compulsive disorder; ODD, oppositional defiant disorder; PTSD, post‐traumatic stress disorder.

#### Child Behavior Checklist

Caregivers in both studies reported on broad child psychopathology symptoms using the CBCL. The 99‐item version for children 1.5–5 years of age (Achenbach & Rescorla, 2000) and the 113‐item version for children 6–18 years of age (Achenbach & Rescorla, 2001) were rated on a 3‐point scale (1 = *Not True*, 2 = *Somewhat or Sometimes True*, 3 = *Very True or Often True*). Syndrome scales (e.g., anxious/depressed) were used to calculate *T*‐scores for broadband internalizing, externalizing, and total problems. Recommended cutoffs were used to indicate whether children exhibited clinical (*T* ≥ 65) versus non‐clinical (*T* < 65) levels along these dimensions.

### Analysis plan

The first two aims of the present investigation were pre‐registered (https://osf.io/gtpdv). All analyses were conducted in Mplus version 8.11 (Muthén & Muthén, 2017) or R version 4.5.0 (R Core Team, 2025). Scripts can be found on GitHub (https://github.com/nicocamacho94/transdx_dim_ec_dep). Data available upon reasonable request and completion of a separate usage agreement.

#### Missing data

Missing data were due to measures being introduced late or not administered to subsets of PCIT‐ED participants or not included in the NOA study. Minimal haphazard missing data were evident. Missing data were treated as missing at random and multiple imputation procedures were implemented (Enders, 2022). Values missing on a measure for all NOA participants would have been imputed using estimated associations between values solely based on PCIT‐ED participant data, so we did not impute them to avoid subsample misrepresentation. Missing KSADS‐EC and CBCL values were not imputed because software limitations precluded conducting a significant portion of the Aim 3 analysis plan using multiply imputed data. Details can be found on Supporting Information S1: Tables S2–S4 and in Supporting Information S1: Figure S4.

#### Aim 1: Re‐evaluation of a multidimensional model of young child depressive symptoms

Factor models were conducted in Mplus using exploratory structural equation models (ESEMs), weighted least squares estimation with mean and variance adjustments, and Target rotation (Morin et al., 2020). Factor loadings (*λ*s) were “meaningful” if significantly different from zero (*p* < .05) and “cardinal” (“primary” in preregistration) if expected to load predominantly onto a factor. Meaningful *λ*s were labeled *small* (|*λ*| < 0.299), *moderate* (|*λ*| = 0.300–0.599), or *large* (|*λ*| ≥ 0.600). *Adequate* model fit was inferred by comparative fit (CFI) and Tucker‐Lewis (TLI) indices ≥0.90 and <0.95, root mean squared error of approximation (RMSEA) ≤ 0.08 and >0.06, and standardized root mean squared residual (SRMR) ≤ 0.10 and >0.08, whereas *close* fit was inferred by CFI/TLI ≥ 0.95, RMSEA ≤ 0.06, and SRMR ≤ 0.08 (Hu & Bentler, 1999; Yuan et al., 2016).

We used a bifactor‐ESEM procedure (Morin, 2023) to test the robustness of the existing multidimensional model of young child depressive symptoms (Hypothesis 1). First, the 3‐CF model of the PFC‐S was estimated (Supporting Information S1: Figure S5A). This model was deemed appropriate if: (A) model fit was adequate, (B) all a priori specified primary *λ*s were meaningful, and (C) cross‐loadings were small to moderate. Next, an orthogonal 3‐bifactor model was estimated (Supporting Information S1: Figure S5B). This model was deemed appropriate and preferred over the 3‐CF model if it evidenced: (D) equivalent or improved model fit, (E) meaningful cardinal *λ*s across all items onto the general factor, and (F) 2+ meaningful cardinal *λ*s for each group factor.

Wording similarities among items with cardinal *λ*s onto the SBA factor have been noted (Camacho et al., 2025). To test whether the SBA factor unduly encompassed method variance associated with such similarities (Hypothesis 2), residual variance correlations among items 1, 3, 5, and 9 were specified using the retained PFC‐S model. Item residual correlations were retained if significant (*p* < .05) and model fit did not worsen. We inferred that the SBA factor reflected a substantive construct if its cardinal items remained meaningfully loaded onto SBA. Otherwise, it was inferred to represent method variance.

Factor reliability, construct replicability, and factor determinacy (FD) estimates were calculated in R using the retained PFC‐S model (Hypothesis 3). Omega (*ω*) was used to test satisfactory unit‐weighted composite score reliability (*ω* ≥ 0.80 for a CF model; *ω*_H_ > 0.75 and *ω*_HS_ ≥ 0.50 for the general and group factors of a bifactor model, respectively) (Rodriguez et al., 2016). Statistic *H* was used to test satisfactory construct replicability (*H* ≥ 0.70) (Rodriguez et al., 2016). Dependable factor scores were indicated by FD values ≥ 0.90 (Gorsuch, 1988; Grice, 2001). To be considered a viable PFC‐S model, at least one factor was expected to meet all relevant reliability criteria.

#### Aim 2: Construct validation of young child depressive symptom dimensions

We planned to estimate in Mplus zero‐order correlations between the reliable PFC‐S factor(s) and (Correlation Model 1) composite mean (sub)scale scores from the ERC, BIS/BAS, and BDI‐II as well as (Correlation Model 2) KSADS‐EC‐based anhedonia severity ratings, BRIEF GEC *T*‐scores, and MC2 Guilt Feelings scores. Validation measures were entered using latent factor placeholders. Steiger's *z*‐tests (Steiger, 1980) were planned to compare the correlations in Model 2 between 2+ reliable PFC‐S factors. See Supporting Information S1: Table S5 for expected correlations (Hypothesis 4) (Camacho et al., 2025).

##### Planned exploratory analyses

Correlations between the reliable PFC‐S factor(s) and child age and sex assigned at birth (female = 0; male = 1) were estimated. All correlations were also estimated using composites calculated for each reliable PFC‐S factor based on mean scores on their respective cardinal items.

##### Preregistration deviations

Mplus required correlations be allowed for all ESEM factors, rather than only those deemed reliable (Muthén & Muthén, 2017). Correlation Model 1 was estimated this way. Only the correlations involving the reliable PFC‐S factor(s) were interpreted. Correlation Model 2 did not converge when specified this way. Here, separate models were specified for each validation measure. Anhedonia severity ratings and GEC *T*‐scores were directly entered into Mplus to avoid model non‐convergence.

#### Aim 3: Young child depressive symptom dimensions as transdiagnostic risk indicators

Reliable factor score(s) from the retained PFC‐S model were extracted from the dataset with missing values using the regression method in Mplus (Muthén & Muthén, 2017). In separate logistic regression models in R, the reliable PFC‐S factor score(s) were used to distinguish between KSADS‐EC‐based diagnostic groups and clinical/non‐clinical levels of CBCL‐based Internalizing, Externalizing, and Total Problems. The accuracy of each significant distinction was estimated in R using receiver operating characteristic curves that depicted the area under the curve, sensitivity, and specificity associated with each optimal PFC‐S factor score cutoff based on the Youden Index (*J*) (Altman & Bland, 1994; Fluss et al., 2005; Perkins & Schisterman, 2006). The likelihoods with which young children would be categorized as belonging to each condition group were also estimated using predicted probabilities associated with PFC‐S factor scores both below and at/above the respective optimal cutoff (Muller & MacLehose, 2014). Greater accuracy was inferred when predicted probabilities approached 0 and 1 for PFC‐S factor scores below and at/above the optimal cutoff, respectively. Supporting Information S1: Table S6 shows hypothesized group distinctions (Hypothesis 5).

## RESULTS

### Aim 1: Re‐evaluation of a multidimensional model of young child depressive symptoms

#### Hypothesis 1

See Supporting Information S1: Table S7. The 3‐CF model of the PFC‐S evidenced *close* fit (CFI = 0.97, TLI = 0.96, SRMR = 0.03), although the RMSEA (0.08) indicated *adequate* fit (Criterion A). It was comprised of well‐defined factors, as cardinal *λ*s were all meaningful (Criterion B) and most were *moderate*‐to‐*large*. Cardinal *λ*s for items 15 and 20 onto EGS were *small*. Items 15 and 20 respectively showed *moderate* cross‐loadings onto SBA and EBD, as did item 5 onto EGS. Items a priori specified to cross‐load onto 2 factors showed *small*‐to‐*moderate λ*s onto them. Most items expected to predominantly load onto one factor also showed 21 meaningful cross‐loadings (18 were *small* and 3 were *moderate*) (Criterion C). The factors were highly correlated with one another (*r*_SBA/EBD_ = .61; *r*_SBA/EGS_ = .60; *r*_EBD/EGS_ = .70; *p*s < .001). The 3‐CF model satisfied Criteria A‐C.

The 3‐bifactor model of the PFC‐S evidenced *close* fit (CFI = 0.98, TLI = 0.98, SRMR = 0.03, RMSEA = 0.06) (Criterion D). It was comprised of a well‐defined general factor (Criterion E). All items loaded meaningfully onto it and showed *moderate*‐to‐*large λ*s. Although group factors were associated with 2+ meaningful cardinal *λ*s (Criterion F), they were less well‐defined (i.e., lower *λ*s) relative to their 3‐CF model counterparts. EGS was the only factor whose cardinal items did not all meaningfully load onto it. Items a priori specified to cross‐load onto 2 factors did not always meaningfully load onto them. Most items expected to predominantly load onto one factor also showed 23 *small* but meaningful cross‐loadings. The 3‐bifactor model satisfied Criteria D–F and was preferred over the 3‐CF model.

#### Hypothesis 2

The 3‐bifactor structure of the PFC‐S with specific item residual correlations evidenced *close* fit (CFI = 0.99, TLI = 0.98, RMSEA = 0.06, SRMR = 0.02). Residual variances between items 1 and 3 (*r* = .26, *p* < .001), 1 and 9 (*r* = .32, *p* < .001), 3 and 9 (*r* = .57, *p* < .001), and 5 and 9 (*r* = .16, *p* = .010) were significantly correlated. Those between items 1 and 5 (*r* = .008, *p* = .947) and 3 and 5 (*r* = .08, *p* = .286) were not. An updated 3‐bifactor model containing only significant item residual correlations (*r*_1/3_ =.24, *r*_1/9_ = .32, *r*_3/9_ = .56, *p*s < .001; *r*_5/9_ = .15, *p* = .008) evidenced *close* fit (CFI = 0.99, TLI = 0.98, RMSEA = 0.06, SRMR = 0.02). Substantial decrements among SBA cardinal *λ*s were evident (Table 2). Items 1 and 10 did not meaningfully load onto SBA and the *λ*s of items 3 and 9 decreased from *moderate* to *small*. Item 5's *λ* increased from *small* to *moderate*. This model was retained (3SRC‐bifactor; Figure 2).

**TABLE 2:** Factor loadings and reliability estimates of the 3SRC‐bifactor model of the PFC‐S.

	General	SBA	EBD	EGS
Items				
1. Interested in playing with other kids^a^	**.723**	.240	−.215	−.164
3. Engages in activities that make him/her happy^a^	**.713**	**.252**	.052	.056
5. Seems less excited about play/activities than other kids	**.849**	**.328**	−.073	.045
9. Enjoys activities and play^a^	**.660**	**.213**	.016	−.019
10. Keeps to him/herself	**.581**	.163	−.179	−.067
17. Seems to be very tired and low energy	**.871**	**−.388**	−.006	−.137
6. Has difficulty sleeping at night	**.605**	−.024	**.528**	−.083
7. Whines/cries	**.493**	.033	**.356**	.056
8. Can't pay attention to games/tasks	**.653**	−.089	**.447**	.167
4. Has a lot of trouble following simple directions	**.584**	−.011	**.528**	.011
11. Pretend plays about scary/sad things	**.591**	−.073	**.227**	.142
23. Seems more irritable/grouchy than other kids his/her age	**.752**	−.264	.054	−.015
14. Is having problems at preschool/daycare	**.823**	−.101	**.261**	.055
21. Used to behave his/her age but now acts younger	**.675**	**.141**	.056	**.184**
22. Has trouble getting along with other kids	**.586**	.113	**.209**	**.126**
2. Appears sad or says s/he feels sad	**.705**	.091	.121	**.246**
13. Seems to lack confidence	**.631**	−.079	.082	**.698**
12. Blames him/herself for things	**.723**	.020	−.032	**.354**
15. Not excited about things other children his/her age find exciting	**.778**	.225	−.086	.017
18. Seems to feel overly guilty	**.664**	.024	−.015	**.644**
19. Seems lonely	**.778**	.067	.058	**.228**
20. Has unusually increased or decreased appetite	**.613**	.082	.253	.061
Reliability estimates				
Omega hierarchical (*ω*_H_ or *ω*_HS_)	.91	.09	.23	.26
Statistic *H*	.96	.23	.58	.66
Factor determinacy (FD)	.99	.33	.65	.80

*Note*: Factor loadings are shown per item and factor. Bolded numbers show expected primary/cardinal loadings, regular black numbers show meaningful loadings (*p* < .05) not expected to be primary/cardinal, and gray numbers show non‐meaningful (*p* ≥ .05) loadings (citation). Item 16 was removed from the present investigation due to prior work demonstrating its low communality with the rest of the PFC‐S items (citation).

Abbreviations: 3‐SRC‐bifactor, bifactor model with 3 group factors and specific item residual correlations; EBD, emotional and behavioral dysregulation; EGS, excessive guilt and sadness; PFC‐S, Preschool Feelings Checklist‐Scale; SBA, Social and Behavioral Anhedonia.

^a^ Reverse‐coded item.

**FIGURE 2:**
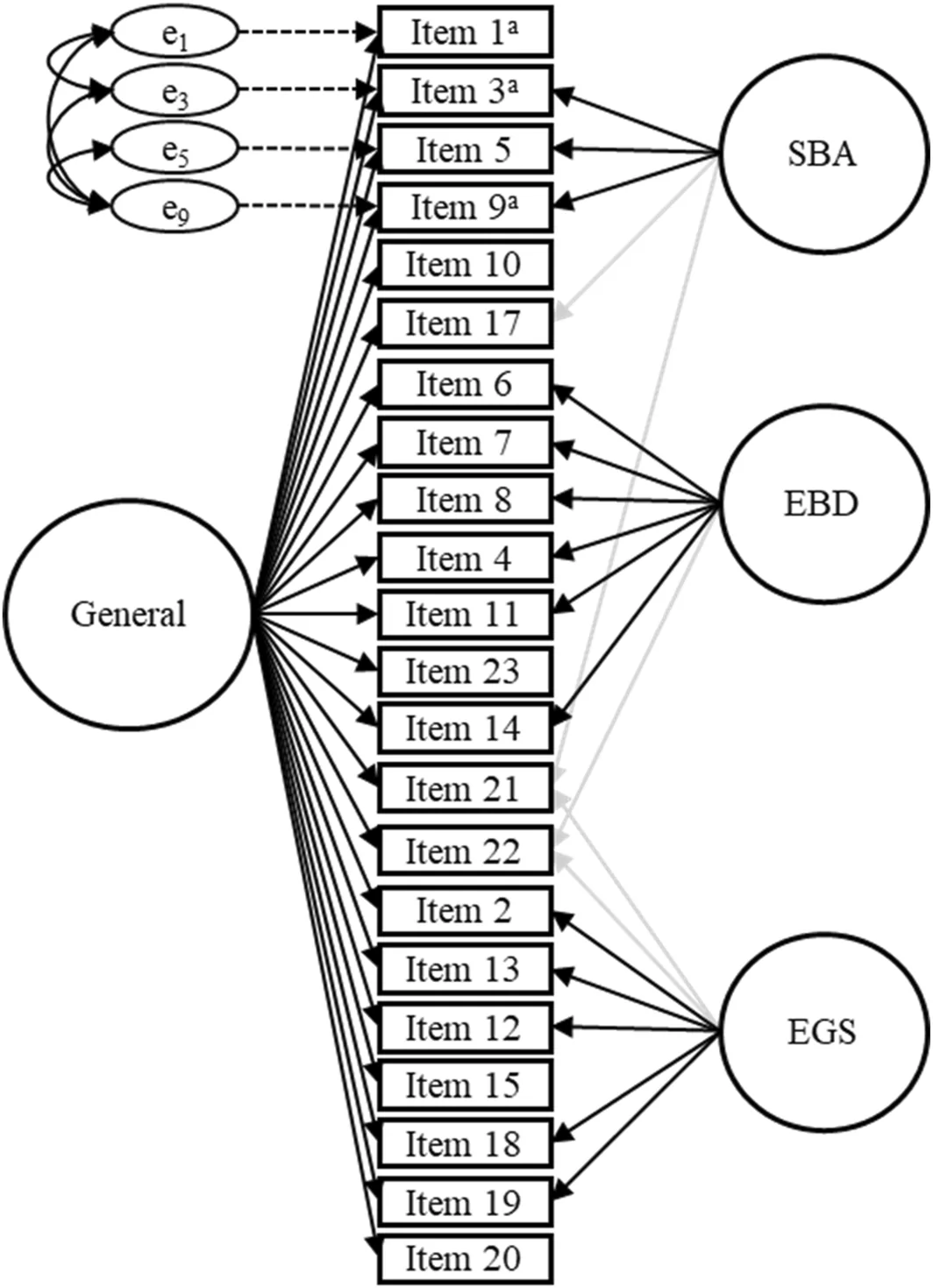
Representation of the 3SRC‐Bifactor model of the PFC‐S. Observed PFC‐S items and latent factors are depicted in rectangles and circles, respectively. Residuals for items 1, 3, 5, and 9 are depicted in ovals and dashed lines at the top left‐hand side of the figure. Curved lines with double‐headed arrows represent correlations. Straight arrows with single‐headed arrows represent factor loadings. Black/dark straight lines depict loadings that remained primary/cardinal per factor as expected, whereas gray/light straight lines depict loadings that cross‐loaded onto 2 factors as expected. Variances, residual terms, and all other loadings in this exploratory structural equation model were not depicted for figure simplicity. Item 16 was removed from the present investigation due to prior work demonstrating its low communality with the rest of the PFC‐S items (citation). 3SRC‐Bifactor, bifactor model with 3 group factors and specific item residual correlations; EBD, emotional and behavioral dysregulation; EGS, excessive guilt and sadness; PFC‐S, Preschool Feelings Checklist‐Scale; SBA, social and behavioral anhedonia. ^a^Reverse‐coded item.

#### Hypothesis 3

Only the general factor of the PFC‐S 3SRC‐bifactor model evidenced satisfactory factor reliability, construct replicability, and FD, precluding the use of the group factors in remaining analyses (Table 2). EGS evidenced relative strengths among the group factors.

### Aim 2: Construct validation of young child depressive symptom dimensions

#### Hypothesis 4

All correlation models evidenced *close* fit (CFIs ≥ 0.98, TLIs ≥ 0.97, RMSEAs ≤ 0.06, SRMRs ≤ 0.03). See Figure 3. As expected, the PFC‐S general factor was positively correlated with LN, BIS, BAS Drive, BDI‐II, anhedonia severity, and GEC; negatively correlated with ER; and not significantly correlated with BAS and BAS Fun‐Seeking. Although we hypothesized no association, we found that the general factor was negatively correlated with BAS Reward Responsiveness. Interestingly, the general factor was not significantly correlated with Guilt Feelings.

**FIGURE 3:**
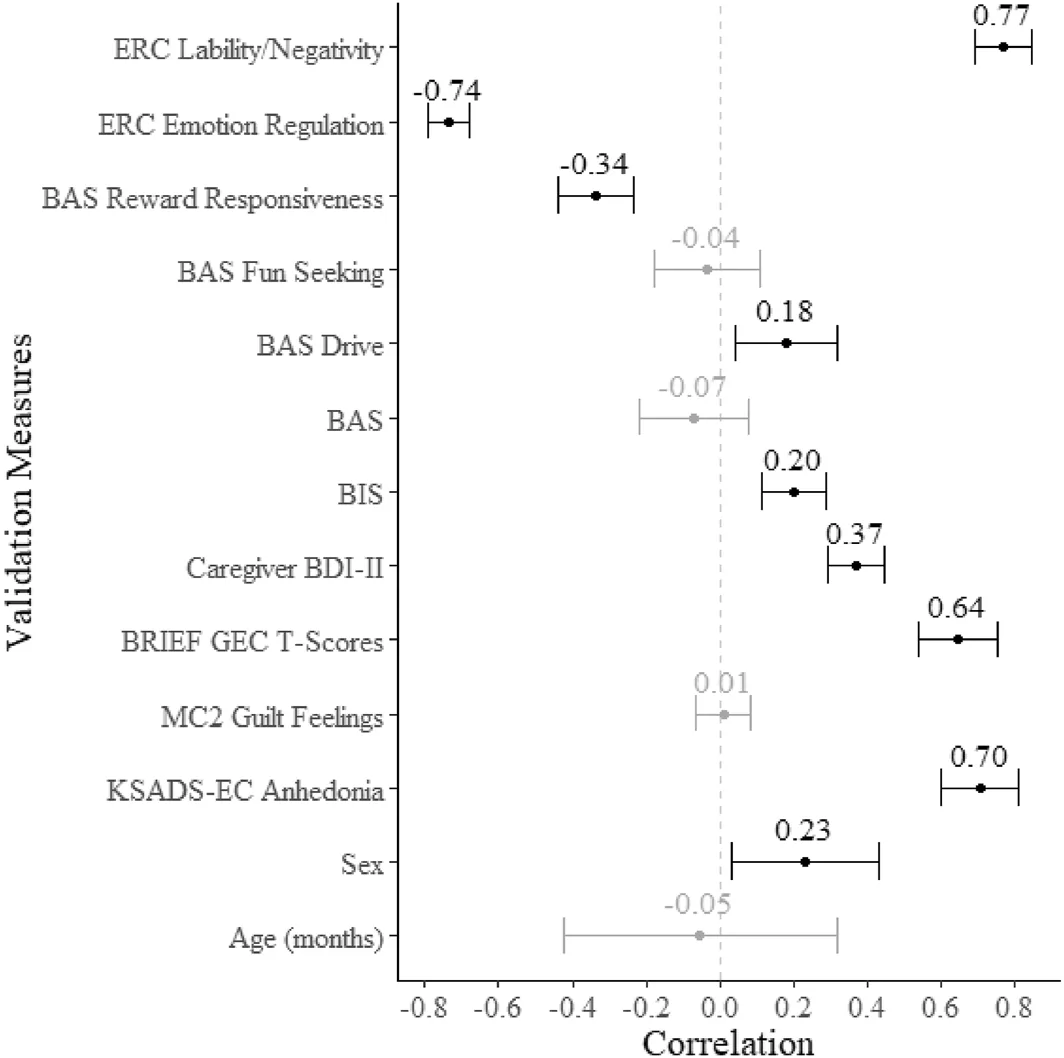
Construct validation of the general factor of the PFC‐S. Significant (*p* < .05) correlations are depicted by black/dark text and shapes, whereas non‐significant (*p* ≥ .05) correlations are depicted by gray/light text and shapes. The dashed line is a visual aid for *r* = 0. Dots depict point estimates and the solid lines depict 95% confidence intervals. While polyserial and point‐biserial correlations were respectively estimated for KSADS‐EC Anhedonia scores and Sex (0 = female, 1 = male), Pearson's correlations were estimated for all other measures. BDI‐II, Beck Depression Inventory–II; BIS/BAS, Behavioral Inhibition/Activation Scales; BRIEF GEC, Behavior Rating Inventory of Executive Function Global Executive Composite; ERC, Emotion Regulation Checklist; KSADS‐EC, Kiddie Schedule for Affective Disorders and Schizophrenia Early Childhood; MC2, My Child 2; PFC‐S, Preschool Feelings Checklist‐Scale.

##### Planned exploratory analyses

The PFC‐S general factor was not significantly correlated with child age. It was positively correlated with sex assigned at birth (0 = female, 1 = male). A unique negative correlation between general factor composite mean scores and child age was found. All other results were consistent with those based on the general factor (Supporting Information S1: Figure S6).

### Aim 3: Young child depressive symptom dimensions as transdiagnostic risk indicators

#### Hypothesis 5

See Table 3. PFC‐S general factor scores significantly distinguished young children based on all group comparisons. However, the accuracy with which they did varied. Children in the “Any Co‐Occurring Diagnoses” group, rather than the “Any Single Diagnosis” group, were more accurately distinguished from children with “No Diagnoses” (Figure 4A). Similarly, children in the MDD/CO+ group, rather than the MDD/CO− group, were more accurately distinguished from children with “No Diagnoses” (Figure 4B). The same pattern emerged when these two MDD groups were distinguished from children in the Any/MDD− group (Figure 4C). The predicted probabilities associated with PFC‐S factor scores below the respective optimal cutoffs were notably higher (i.e., less accurate) when children with MDD were distinguished from those with Any/MDD− groups versus those with “No Diagnoses.” Although relatively accurate across all other metrics, the distinction between children in the MDD/ANX− and ANX/MDD− groups (Figure 4D) resulted in the highest predicted probability (i.e., least accurate) associated with PFC‐S factor scores below the respective optimal cutoff.

**TABLE 3:** Metrics associated with psychopathology group distinctions based on PFC‐S general factor scores.

	Metrics				
	Log odds [95% CI]	AUC	Youden index (sensitivity, specificity)	Pred. prob. below [95% CI]	Pred. prob. at/above [95% CI]
KSADS‐based group comparisons (reference vs. condition)					
No versus any single diagnosis	1.76 [1.28, 2.29]	0.81	0.46 (0.66, 0.80)	0.22 [0.16, 0.30]	0.69 [0.58, 0.78]
No versus any co‐occurring diagnoses	3.60 [2.92, 4.41]	0.96	0.81 (0.92, 0.89)	0.12 [0.08, 0.19]	0.93 [0.89, 0.96]
No diagnoses versus MDD/CO−	2.65 [1.97, 3.46]	0.89	0.57 (0.76, 0.81)	0.13 [0.08, 0.20]	0.66 [0.54, 0.76]
No diagnoses versus MDD/CO+	4.23 [3.39, 5.26]	0.97	0.83 (0.94, 0.89)	0.09 [0.05, 0.16]	0.93 [0.89, 0.96]
Any/MDD− versus MDD/CO−	1.93 [1.15, 2.87]	0.83	0.41 (0.76, 0.65)	0.43 [0.28, 0.59]	0.81 [0.69, 0.89]
Any/MDD− versus MDD/CO+	3.36 [2.43, 4.51]	0.93	0.69 (0.91, 0.77)	0.42 [0.28, 0.57]	0.96 [0.92, 0.98]
ANX/MDD− versus MDD/ANX−	2.89 [2.02, 3.94]	0.92	0.64 (0.82, 0.82)	0.57 [0.43, 0.70]	0.97 [0.92, 0.99]
CBCL‐based group comparisons (non‐clinical vs. clinical)					
Internalizing problems	2.24 [1.87, 2.65]	0.88	0.59 (0.89, 0.71)	0.10 [0.07, 0.14]	0.67 [0.61, 0.72]
Externalizing problems	1.74 [1.42, 2.08]	0.84	0.54 (0.88, 0.67)	0.09 [0.06, 0.13]	0.57 [0.51, 63]
Total problems	2.64 [2.21, 3.12]	0.91	0.65 (0.88, 0.77)	0.10 [0.07, 0.14]	0.73 [0.67, 0.78]

*Note*: Log odds were estimated from logistic regression models. All other metrics were based on receiver operating characteristic curve analyses. While children were allowed to be included in multiple groups, group comparisons were mutually exclusive (i.e., it was not possible for the same child to show up in both the Reference and Condition groups of a given comparison).

Abbreviations: ANX, 1+ anxiety diagnosis; ANX–, no anxiety diagnoses; Any, any diagnoses; AUC, area under the curve; CBCL, Child Behavior Checklist; CI, confidence interval; CO−, no co‐occurring diagnoses; CO+, with co‐occurring diagnoses; KSADS, Kiddie Schedule for Affective Disorders and Schizophrenia; MDD, major depressive disorder; MDD−, no MDD; PFC‐S, Preschool Feelings Checklist‐Scale; Pred. prob., predictive probabilities.

**FIGURE 4:**
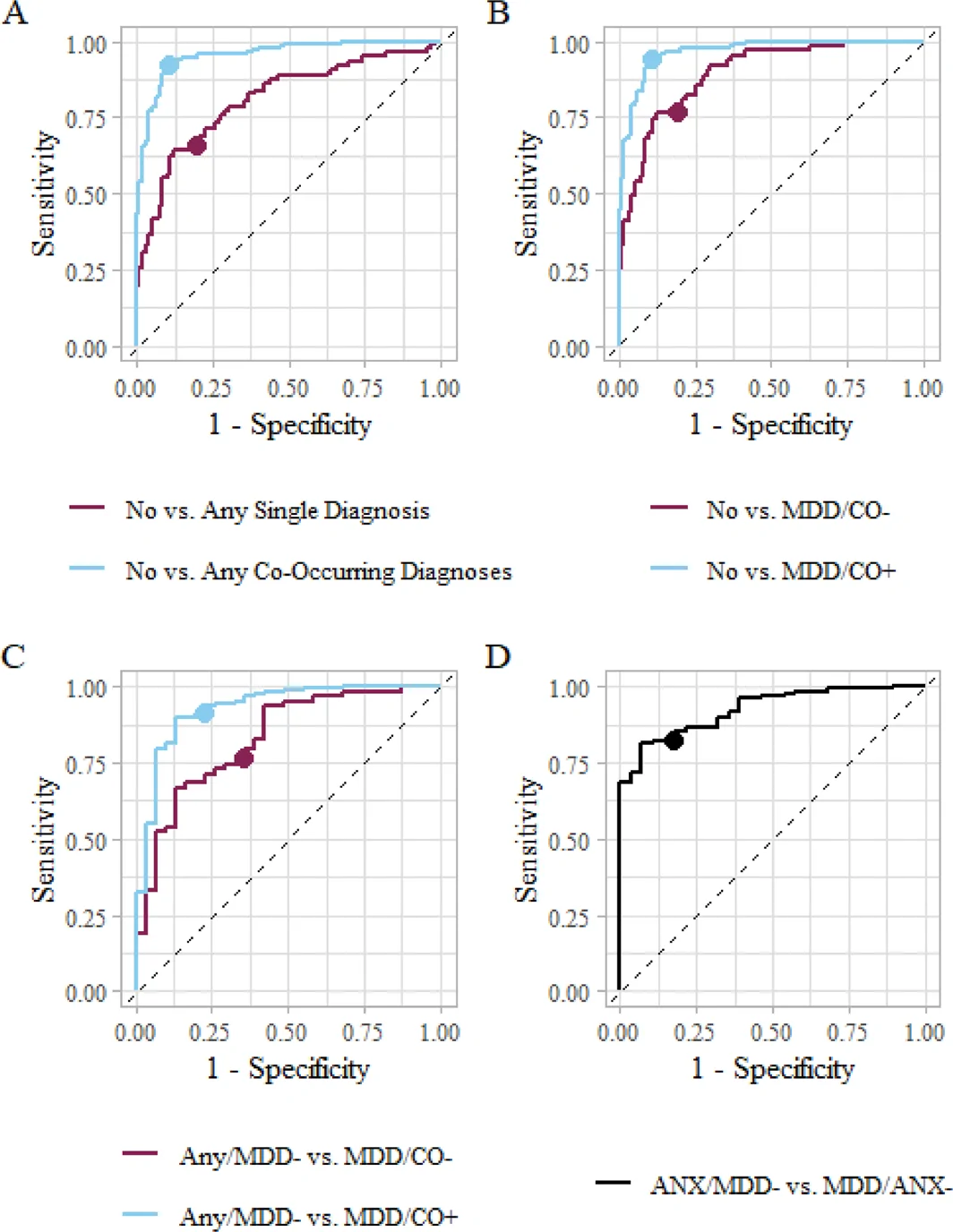
Receiver operating characteristic curves associated with KSADS‐EC diagnostic group distinctions based on PFC‐S general factor scores. Specifically, (A) children with no diagnoses were distinguished from those with any single diagnosis and those with any co‐occurring diagnoses, (B) children with no diagnoses were distinguished from those with MDD with and without co‐occurring diagnoses, (C) children with any diagnosis other than MDD were distinguished from those with MDD with and without co‐occurring diagnoses, and (D) children with anxiety but not MDD diagnoses were distinguished from those with MDD but not anxiety diagnoses. Dashed lines depict the line associated with a random (50% chance) classifier. Solid lines depict receiver operating characteristic curves for the group comparison associated with the color of the line. Dots depict the optimal PFC‐S General Factor score cutoff for the specific group comparison based on the Youden Index. ANX, 1+ anxiety diagnosis; ANX−, no anxiety diagnoses; Any, any diagnoses; CO−, no co‐occurring diagnoses; CO+, with co‐occurring diagnoses; KSADS‐EC, Kiddie Schedule for Affective Disorders and Schizophrenia Early Childhood; MDD, major depressive disorder; MDD–, no MDD; PFC‐S, Preschool Feelings Checklist‐Scale.

As expected, PFC‐S general factor scores significantly distinguished young children with Clinical versus Non‐Clinical levels on all three broadband dimensional psychopathology spectra (Figure 5). Distinctions based on clinical concern for Total Problems was relatively more accurate than those based on Internalizing Problems, which themselves were more accurate than those based on Externalizing Problems.

**FIGURE 5:**
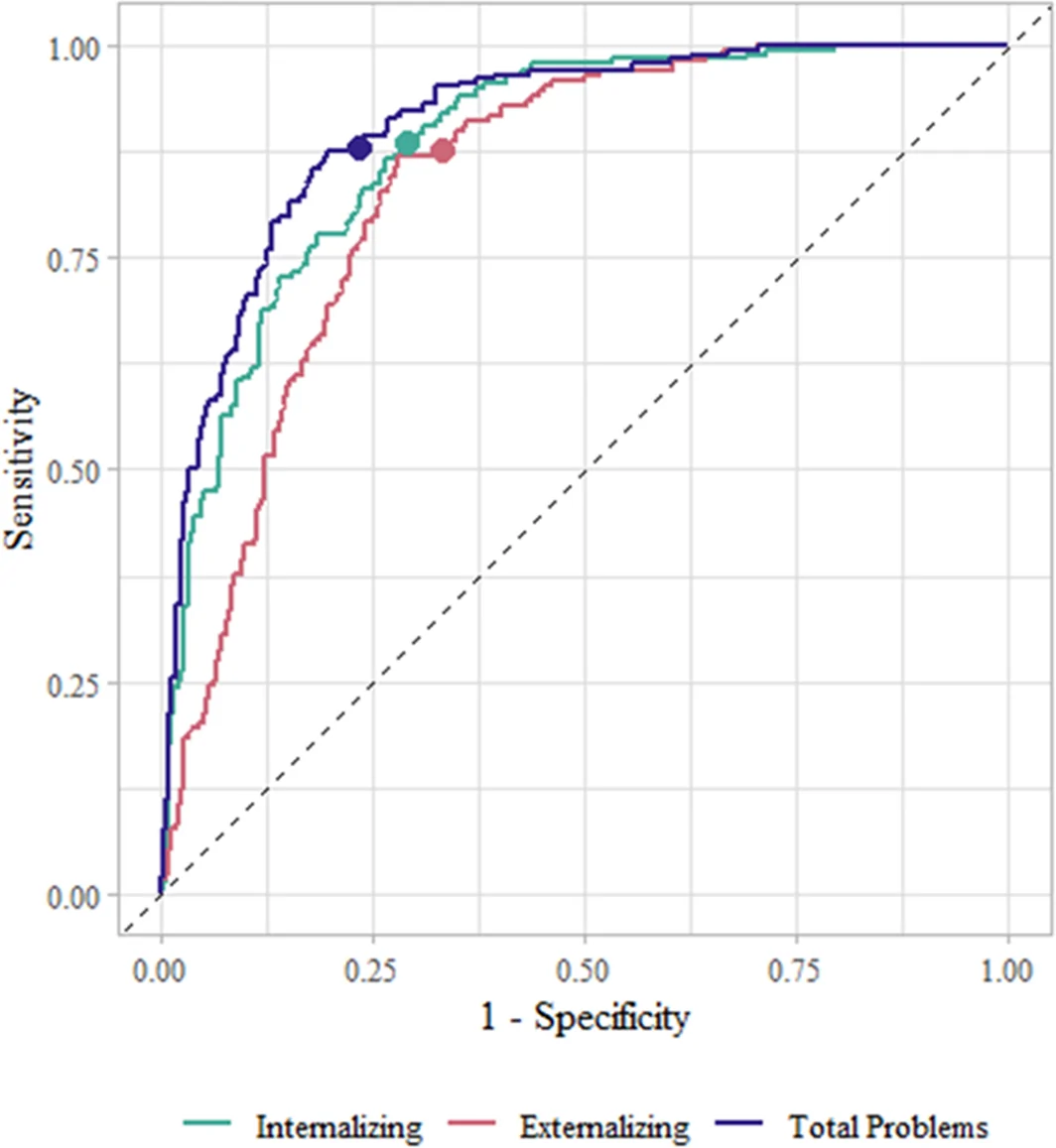
Receiver operating characteristic curves associated with CBCL clinical group distinctions based on PFC‐S general factor scores. The dashed line depicts the line associated with a random (50% chance) classifier. Solid lines depict receiver operating characteristic curves for the comparisons of children grouped by non‐clinical versus clinical *T*‐scores on the broadband psychopathology dimensions specified by line color. Dots depict the optimal PFC‐S General Factor score cutoff for the specific group comparison based on the Youden Index. CBCL, Child Behavior Checklist; PFC‐S, Preschool Feelings Checklist‐Scale.

## DISCUSSION

Using a large sample of young children aged 3–7 years and enriched for internalizing symptoms, we investigated the psychometric properties of the PFC‐S and its utility in indicating transdiagnostic risk for psychopathology. We found that while young child depressive symptoms are multidimensional, they are most robustly represented by a single PFC‐S general factor associated with potential challenges in reward‐related processing, affective and behavioral dysregulation, and executive functioning. This factor was most accurately indicative of diagnostic co‐occurrence, especially among young children with MDD, and clinical concern along a global index of psychopathology. These findings conceptually replicated and extended prior work (Camacho et al., 2025) in an independent sample and support the PFC‐S as relevant to early‐emerging depression as well as a transdiagnostic tool.

In Aim 1, we conceptually replicated the 3‐CF model of the PFC‐S (Camacho et al., 2025), which represented depressive symptom dimensions encompassing SBA, EBD, and EGS. However, we found that the SBA factor may represent wording similarities among its cardinal items, rather than a substantive construct. The three group factors of the PFC‐S did not meet satisfactory reliability criteria when modeled alongside a robust and reliable general factor. The identification of this general factor supports prior uses of the PFC‐S (Fowler et al., 2021; Fowler & Gaffrey, 2022; Gaffrey, Barch, et al., 2018; Heaton et al., 2023; Luby et al., 2020, 2021; Whalen et al., 2020) and bifactor models of depressive symptoms (Gomez & McLaren, 2015; Heinrich et al., 2020; Kóbor et al., 2013; Zhao et al., 2024). Notably, this general factor was identified within a multidimensional model, suggesting that young child depressive symptoms are not simply unidimensional (Fried et al., 2022). Instead, they encompass various phenotypes previously associated with distinct neural, psychological, and environmental correlates (Donohue et al., 2020; Luby, Agrawal, et al., 2018). For example, maladaptive guilt is associated with dorsomedial prefrontal cortex thinning not associated with other depressive symptoms in preschoolers (Donohue et al., 2020). Our findings support the PFC‐S as a general measure of depressive symptom dimensionality in young children and underscore the importance of modeling more specific underlying dimensions in future work. Continued psychometric investigations of the PFC‐S may result in the reliable measurement of general and specific young child depressive symptom dimensions.

Although general factors often recapitulate respective group factors in bifactor models, this may not be the case for depressive symptoms (Watts et al., 2019). This was reflected in the results of Aim 2, which showed that the general factor of the PFC‐S 3SRC‐bifactor model was related to reward‐related processing (i.e., anhedonia, reward responsiveness), affective and behavioral dysregulation (e.g., emotional lability, driven goal pursuit, punishment avoidance), and executive dysfunction, but not negative self‐evaluative or self‐conscious feelings (e.g., shame/guilt), fun‐seeking, or general approach behavior. The general factor not significantly correlating with Guilt Feelings may be explained by the fact that guilt‐related symptoms on the PFC‐S (i.e., items 12, 13, and 18) remained at least moderately loaded onto EGS and that EGS showed relative strengths in construct replicability and FD. Further, the correlations of the PFC‐S general factor in Correlation Model 1 were most closely aligned with those expected of the SBA and EBD factors (Camacho et al., 2025). Therefore, the general factor seems to intersect blunted reward‐related processing and affective and behavioral dysregulation. This interpretation is supported by work showing that links between these constructs are already apparent in preschoolers (Qu et al., 2013) and persist well into adulthood (Dombrovski et al., 2015; Peterson et al., 2022). Interestingly, executive function is known to support processes involved in both emotional/behavioral regulation and reward learning/pursuit (Blair, 2003; Calkins & Marcovitch, 2010; Hughes et al., 2023; Pizzagalli, 2014). Furthermore, young child depressive symptomatology as measured by the PFC‐S has been linked with the structure and function of frontal, striatal, and limbic brain regions implicated in reward processing and emotion (dys)regulation (Fowler & Gaffrey, 2022; Gaffrey, Barch, et al., 2018; Rappaport et al., 2020). Because reward‐related, affective, and executive functioning processes are relevant across many forms of psychopathology (Beauchaine & Cicchetti, 2019; Snyder et al., 2019; Zald & Treadway, 2017), the PFC‐S general factor potentially indicates transdiagnostic risk for psychopathology.

In Aim 3, we found that the PFC‐S general factor indicated the presence of psychiatric disorders in young children and was particularly accurate as an indicator of disorder co‐occurrence, especially if children presented with MDD. We also found that while the general factor accurately indicated clinical elevations in internalizing and externalizing psychopathology, it was most accurate as an indicator of a general dimension of psychopathology (i.e., p‐factor) (Caspi et al., 2024). These findings align with prior work showing that a checklist version of the PFC‐S accurately distinguished depressed preschoolers with co‐occurring disorders from those with no diagnoses at all (Luby, Heffelfinger, et al., 2004). They are also supported by work using the checklist version of the PFC‐S as an early identification tool for various internalizing and externalizing problems across childhood and adolescence (Silver, Barch, et al., 2021; Silver, Olino, et al., 2021). Diagnostic co‐occurrence is reflective of an increased liability for general psychopathology (Caspi et al., 2024; Kotov et al., 2017). That the PFC‐S general factor was closely linked to diagnostic co‐occurrence, especially when inclusive of MDD, is further evidence of its transdiagnostic utility. These results corroborate with evidence from prior work with an independent sample of preschoolers noting the relevance of early emerging depressive symptom elevations across a wide range of psychiatric problems including Separation Anxiety, Social Phobia, ADHD, CD, and ODD (Sterba et al., 2007). These findings further support the PFC‐S general factor as a transdiagnostic dimension of psychopathology.

Notably, the PFC‐S general factor was capable of distinguishing young children who presented with MDD from those in both the Any/MDD− and ANX/MDD− groups. However, these distinctions were made with less accuracy than would be expected if the factor reflected a phenotype unique to depression. Even so, these distinctions suggest that the PFC‐S general factor characterizes challenges central to young child depression. Although the PFC‐S includes symptoms relevant across multiple disorders, it primarily represents young child MDD symptoms (Camacho et al., 2025). Therefore, it is limited in the extent to which it reflects the full range of young child psychiatric symptomatology (Caspi et al., 2024). Conversely, this may also prove useful in distinguishing psychopathology with distinct etiologies, evidenced by the general factor distinguishing between young children with MDD or anxiety disorders in the present investigation. Anxiety may be more closely related to fear and intolerance of uncertainty (Ryan et al., 2025) than to the reward‐related and executive dysfunctions relevant in depression (Pizzagalli, 2014) and other forms of psychopathology (Zald & Treadway, 2017). Ultimately, the PFC‐S general factor showed optimal utility as a screener for compounded forms of psychopathology and emerging capacity to distinguish children with MDD or anxiety disorders.

### Limitations

The present investigation has limitations. The sample's demographics limit the relevance of our findings to non‐maternal caregivers and underrepresented racial/ethnic and socioeconomic groups of young children. Relatedly, our findings should be interpreted as predominantly relevant to young children with elevated internalizing problems. For example, reward‐related dysfunction may be more salient to caregivers in samples of children with greater depressive symptom acuity (Luby, Mrakotsky, et al., 2004). The use of multiple imputation precluded the use of significance tests of PFC‐S factor model fit comparisons in Aim 1. Nevertheless, PFC‐S factor models were compared following a validated approach for ESEMs (Morin, 2023). Measurement invariance tests were not conducted on the 3SRC‐bifactor model given the large sample size requirements for such analyses (Bader et al., 2022). The construct validation of the PFC‐S general factor based solely on caregiver‐reports may have led to inflated correlations among measures in Aim 2. However, such inflations were mitigated by the estimation of these correlations directly from the 3SRC‐bifactor model (DeShon, 1998). The high prevalence of MDD and co‐occurrence of MDD and DBDs precluded the creation of more specific group comparisons in Aim 3, limiting the extent to which the PFC‐S general factor could be tested as a specific indicator of MDD. Finally, the identified PFC‐S factor score cutoffs and related log odds may not represent those in the general population, due to the overrepresentation of psychopathology in the present sample.

### Future directions

The present investigation conceptually replicated and extended prior work on the psychometric properties of the PFC‐S (Camacho et al., 2025). Future studies can build upon this work by improving PFC‐S symptom descriptions and using increasingly confirmatory factor analytic approaches, possibly resulting in the identification of models with more robust group factors (e.g., anhedonia). Second, multi‐method construct validation is needed to bolster and reify PFC‐S factor interpretations. For example, caregiver reports on the PFC‐S should be cross‐validated with task‐based observations (e.g., Stars‐in‐Jars) (Briggs‐Gowan et al., 2014) and child self‐reports (e.g., MacArthur Story Stem Battery) (Emde et al., 2003) that assess reward‐related and executive functioning processes. Neuroimaging could also be used to identify neural markers implicated in these processes (e.g., cortico‐striatal‐thalamic circuit of the salience network and event‐related potentials of reward responses) (Belden et al., 2016; Peters et al., 2016). Third, longitudinal studies involving the PFC‐S could help inform its structural stability across development and elucidate associations with early emerging temperament traits, such as neuroticism (Compas et al., 2010) and later forms of psychopathology and risky behaviors (e.g., suicidal ideation/attempts) (Hennefield et al., 2024). Fourth, the PFC‐S should be studied alongside other psychopathology measures with similar breadth and depth to facilitate the identification of transdiagnostic dimensions in young children. Candidate measures include but are not limited to the Preschool Anxiety Scale (Zhou et al., 2024), Overcontrol in Youth Checklist (Gilbert et al., 2020), and Multidimensional Assessment of Preschool Disruptive Behavior (Wakschlag et al., 2014). Finally, conducting future work involving the PFC‐S in large samples of young children characterized by diversity in racial/ethnic, socioeconomic, diagnostic, and informant identities will help clarify for whom and under what conditions specific depression‐related dimensions are most salient (Olino, 2020).

## CONCLUSION

Findings from the present investigation suggest that the PFC‐S predominantly and most reliably assesses a transdiagnostic dimension of young child reward‐related and executive dysfunction indicative of diagnostic co‐occurrence, especially among those with MDD, and clinical concern along a global index of psychopathology. These findings help to situate young child depression‐related symptoms within a broader psychiatric nosology and inform the emerging use of the PFC‐S both for research and clinical purposes.

## AUTHOR CONTRIBUTIONS

**Nicolas L. Camacho**: Conceptualization; investigation; writing—original draft; methodology; visualization; writing—review and editing; formal analysis; resources; software; data curation; funding acquisition. **Kirsten E. Gilbert**: Conceptualization; investigation; funding acquisition; writing—review and editing; data curation; resources. **Rebecca Tillman**: Data curation; investigation; validation; resources; writing—review and editing. **Joan L. Luby**: Investigation; funding acquisition; resources; writing—review and editing. **Michael S. Gaffrey**: Conceptualization; investigation; supervision; project administration; resources; writing—review and editing.

## CONFLICT OF INTEREST STATEMENT

The authors declare no conflicts of interest.

## ETHICAL CONSIDERATIONS

Caregivers provided informed consent and children provided assent. The Duke University Institutional Review Board approved data sharing and secondary analysis (September 26, 2024; Protocol: 2024‐0484) via a data usage agreement with Washington University School of Medicine (April 30, 2024; Agreement ID: P24‐07100). Study procedures were conducted in accordance with the Declaration of Helsinki.

## Supporting information

Supporting Information S1

## Data Availability

The data that support the findings of this study are available on request from the corresponding author. The data are not publicly available due to privacy or ethical restrictions.
