# Supplementary material for: Establishing a transdiagnostic dimension of early childhood depressive symptoms using the Preschool Feelings Checklist‐Scale
Source: JCPP Adv. 2026 Oct 9:e70152. Online ahead of print. doi: 10.1002/jcv2.70152 (PMC13650965; doi:10.1002/jcv2.70152)
Supplement: Supplementary file 1 [file JCV2-9999-e70152-s001.docx]

**Establishing a Transdiagnostic Dimension of Early Childhood Depressive Symptoms using the Preschool Feelings Checklist-Scale**

**Supporting Information**

**Table of Contents**

Figure S1. Study Recruitment Strategies, Inclusion/Exclusion Criteria, and Participation Dates

Table S1. The Preschool Feelings Checklist-Scale (PFC-S)

Figure S2. Item-level Data of the Preschool Feelings Checklist-Scale (PFC-S)

Figure S3. Visualization of the Overlap in KSADS-EC Diagnostic Types

Table S2. Missing Data on the Preschool Feelings Checklist-Scale (PFC-S)

Table S3. Missing Data on the Validation Measures

Table S4. Missing Data on CBCL Dimensions and KSADS-EC Diagnoses

Figure S4. Predictor Matrix Used in Multiple Imputation of Missing Data

Figure S5. Hypothesized Factor Models of the Preschool Feelings Checklist–Scale (PFC-S)

Table S5. Hypothesized Correlations between PFC-S Factors and Construct Validation Measures

Table S6. Hypothesized Group Distinctions based on PFC-S Factor Score Optimal Cutoffs

Table S7. Factor Loadings of the 3-CF and 3-Bifactor Models of the PFC-S

Figure S6. Construct Validation of the PFC-S General Factor Composite Mean Scores

Figure S1. Study Recruitment Strategies, Inclusion/Exclusion Criteria, and Participation Dates

*Note*. PCIT-ED = Parent-Child Interaction Therapy–Emotion Development, NOA = Neurodevelopment of Overcontrol and Anxiety, MDD = Major Depressive Disorder, IQ = Intelligence Quotient.

Table S1. The Preschool Feelings Checklist-Scale (PFC-S)

Items

1. Interested in playing with other kids ^a^

2. Appears sad or says s/he feels sad

3. Engages in activities that make him/her happy ^a^

4. Has a lot of trouble following simple directions

5. Seems less excited about play/activities than other kids

6. Has difficulty sleeping at night

7. Whines/cries

8. Can’t pay attention to games or tasks

9. Enjoys activities and play ^a^

10. Keeps to him/herself

11. Pretend plays about scary/sad things

12. Blames him/herself for things

13. Seems to lack confidence

14. Is having problems at preschool/daycare

15. Not excited about things that other children his/her age find exciting

16. Isn’t upset by things that other children his/her age find upsetting

17. Seems to be very tired and has low energy

18. Seems to feel overly guilty

19. Seems lonely

20. Has unusually increased or decreased appetite

21. Used to behave his/her age but now acts younger

22. Has trouble getting along with other kids

23. Seems more irritable/grouchy than other kids his/her age

*Note*. The PFC-S uses a 5-point scale (0 = *Never*, 1 = *Rarely*, 2 = *Sometimes*, 3 = *Often*, 4 = *Most of the time*). Item 16 was removed from the present investigation due to prior work demonstrating its low communality with the rest of the PFC-S items (citation).

^a^ Reverse-coded item.

Figure S2. Item-level Data of the Preschool Feelings Checklist-Scale (PFC-S)

*Note*. The PFC-S uses a 5-point scale (0 = *Never*, 1 = *Rarely*, 2 = *Sometimes*, 3 = *Often*, 4 = *Most of the time*). Bars represent the number of times a certain response option was chosen per item by caregivers in each study for the children in the present sample. PCIT-ED = Parent-Child Interaction Therapy–Emotion Development and NOA = Neurodevelopment of Overcontrol and Anxiety.

Figure S3. Visualization of the Overlap in KSADS-EC Diagnostic Types

*Note*. Numbers represent the number of children with the combination of disorders specified using the overlapping ellipses. MDD = Major Depressive Disorder, DBDs = Disruptive Behavior Disorders (i.e., Oppositional Defiant Disorder, Conduct Disorder, and Attention-Deficit/Hyperactivity Disorder), Other = Mania/Hypomania, Obsessive-Compulsive Disorder, and/or Post-Traumatic Stress Disorder.

Table S2. Missing Data on the Preschool Feelings Checklist-Scale (PFC-S)

| Item | PCIT-ED  *n* (%) | NOA  *n* (%) | Full Sample  *n* (%) | Reasons for Missingness |
| --- | --- | --- | --- | --- |
| 1 | 0 (0) | 0 (0) | 0 (0) | - |
| 2 | 1 (0.27) | 0 (0) | 1 (.18) | Haphazard missingness |
| 3 | 0 (0) | 0 (0) | 0 (0) | - |
| 4 | 1 (0.27) | 0 (0) | 1 (.18) | Haphazard missingness |
| 5 | 0 (0) | 0 (0) | 0 (0) | - |
| 6 | 1 (0.27) | 0 (0) | 1 (.18) | Haphazard missingness |
| 7 | 0 (0) | 0 (0) | 0 (0) | - |
| 8 | 1 (0.27) | 0 (0) | 1 (.18) | Haphazard missingness |
| 9 | 0 (0) | 0 (0) | 0 (0) | - |
| 10 | 0 (0) | 0 (0) | 0 (0) | - |
| 11 | 0 (0) | 0 (0) | 0 (0) | - |
| 12 | 0 (0) | 0 (0) | 0 (0) | - |
| 13 | 0 (0) | 0 (0) | 0 (0) | - |
| 14 | 3 (0.80) | 0 (0) | 3 (.55) | Haphazard missingness |
| 15 | 0 (0) | 0 (0) | 0 (0) | - |
| 16 | 0 (0) | 0 (0) | 0 (0) | - |
| 17 | 0 (0) | 0 (0) | 0 (0) | - |
| 18 | 1 (0.27) | 0 (0) | 1 (.18) | Haphazard missingness |
| 19 | 0 (0) | 0 (0) | 0 (0) | - |
| 20 | 1 (0.27) | 0 (0) | 1 (.18) | Haphazard missingness |
| 21 | 0 (0) | 0 (0) | 0 (0) | - |
| 22 | 1 (0.27) | 0 (0) | 1 (.18) | Haphazard missingness |
| 23 | 0 (0) | 0 (0) | 0 (0) | - |

*Note*. PCIT-ED = Parent-Child Interaction Therapy–Emotion Development Study and NOA = Neurodevelopment of Overcontrol and Anxiety Study. Haphazard missingness specifies that data were missing due to various reasons not under the control of the study teams (e.g., caregivers mistakenly skipping questions).

Table S3. Missing Data on the Validation Measures

| Measure | PCIT-ED  *n* (%) | NOA  *n* (%) | Full Sample  *n* (%) | Reasons for Missingness |
| --- | --- | --- | --- | --- |
| ERC Lability/Negativity | 10 (2.67) | 2 (1.16%) | 12 (2.2) | Haphazard missingness |
| ERC Emotion Regulation | 9 (2.41) | 1 (0.58%) | 10 (1.83) | Haphazard missingness |
| Behavioral Inhibition Scale | 49 (13.10) | 173 (100) | 222 (40.59) | Introduced late to the PCIT-ED study  Not collected at all for NOA |
| Behavioral Activation Scale | 46 (12.30) | 173 (100) | 219 (40.04) | Introduced late to the PCIT-ED study  Not collected at all for NOA |
| BAS Reward Responsiveness | 47 (12.57) | 173 (100) | 220 (40.22) | Introduced late to the PCIT-ED study  Not collected at all for NOA |
| BAS Drive | 48 (12.83) | 173 (100) | 221 (40.40) | Introduced late to the PCIT-ED study  Not collected at all for NOA |
| BAS Fun-Seeking | 46 (12.30) | 173 (100) | 219 (40.04) | Introduced late to the PCIT-ED study  Not collected at all for NOA |
| BDI-II Depressive Severity | 0 (0) | 0 (0) | 0 (0) | - |
| KSADS-EC Anhedonia Severity | 60 (16.04) | 3 (1.73%) | 63 (11.52) | Not completed by community controls in PCIT-ED  Some haphazard missingness in PCIT-ED  Unreliable or incomplete diagnostic consensus in NOA |
| BRIEF GEC | 37 (9.89) | 7 (4.05%) | 44 (8.04) | Not collected for healthy controls in PCIT-ED  Haphazard missingness for NOA |
| MC2 Guilt Feelings | 97 (25.94) | 173 (100) | 270 (49.36) | Haphazard missingness in PCIT-ED  Not collected for all control participants in PCIT-ED  Not collected at all for NOA |

*Note*. PCIT-ED = Parent-Child Interaction Therapy–Emotion Development Study, NOA = Neurodevelopment of Overcontrol and Anxiety Study, ERC = Emotion Regulation Checklist, BAS = Behavioral Activation Scale, BDI-II = Beck Depression Inventory–II, KSADS-EC = Kiddie Schedule for Affective Disorders and Schizophrenia Early Childhood, BRIEF GEC = Behavior Rating Inventory of Executive Function Global Executive Composite, and MC2 = My Child 2. Haphazard missingness specifies that data were missing due to various reasons not under the control of the study teams (e.g., caregivers mistakenly skipping questions).

Table S4. Missing Data on CBCL Dimensions and KSADS-EC Diagnoses

| Measure | PCIT-ED  *n* (%) | NOA  *n* (%) | Full Sample  *n* (%) | Reasons for Missingness |
| --- | --- | --- | --- | --- |
| Internalizing Problems | 43 (11.50) | 1 (0.58) | 44 (8.04) | Not collected from healthy controls in PCIT-ED  Haphazard missingness |
| Externalizing Problems | 43 (11.50) | 1 (0.58) | 44 (8.04) | Not collected from healthy controls in PCIT-ED  Haphazard missingness |
| Total Problems | 43 (11.50) | 1 (0.58) | 44 (8.04) | Not collected from healthy controls in PCIT-ED  Haphazard missingness |
| Major Depressive Disorder | 59 (15.78) | 3 (1.73) | 62 (11.33) | Not collected from community controls in PCIT-ED  Haphazard missingness in PCIT-ED  Unreliable or incomplete diagnostic consensus in NOA |
| Mania/Hypomania | 112 (29.95) | 4 (2.31) | 116 (21.21) | Not collected from control participants in PCIT-ED  Incomplete diagnostic consensus for NOA  Haphazard missingness |
| Separation Anxiety | 106 (28.34) | 3 (1.73) | 109 (19.93) | Not collected from control participants in PCIT-ED  Incomplete diagnostic consensus for NOA  Haphazard missingness |
| Social Anxiety/Phobia | 115 (30.75) | 3 (1.73) | 118 (21.57) | Not collected from control participants in PCIT-ED  Incomplete diagnostic consensus for NOA  Haphazard missingness |
| Specific Phobia | 115 (30.75) | 3 (1.73) | 118 (21.57) | Not collected from control participants in PCIT-ED  Incomplete diagnostic consensus for NOA  Haphazard missingness |
| Generalized Anxiety | 115 (30.75) | 3 (1.73) | 118 (21.57) | Not collected from control participants in PCIT-ED  Incomplete diagnostic consensus for NOA  Haphazard missingness |
| Obsessive-Compulsive Disorder | 115 (30.75) | 3 (1.73) | 118 (21.57) | Not collected from control participants in PCIT-ED  Incomplete diagnostic consensus for NOA  Haphazard missingness |
| Attention-Deficit/Hyperactivity Disorder | 115 (30.75) | 5 (2.89) | 120 (21.94) | Not collected from control participants in PCIT-ED  Incomplete diagnostic consensus for NOA  Haphazard missingness |
| Oppositional Defiant Disorder | 117 (31.28) | 7 (4.05) | 124 (22.67) | Not collected from control participants in PCIT-ED  Incomplete diagnostic consensus for NOA  Haphazard missingness |
| Conduct Disorder | 121 (32.35) | 7 (4.05) | 128 (23.40) | Not collected from control participants in PCIT-ED  Incomplete diagnostic consensus for NOA  Haphazard missingness |
| Post-Traumatic Stress Disorder | 121 (32.35) | 3 (1.73) | 124 (22.67) | Not collected from control participants in PCIT-ED  Incomplete diagnostic consensus for NOA  Haphazard missingness |

*Note*. KSADS-EC = Kiddie Schedule for Affective Disorders and Schizophrenia Early Childhood, PCIT-ED = Parent-Child Interaction Therapy–Emotion Development Study and NOA = Neurodevelopment of Overcontrol and Anxiety Study. Haphazard missingness specifies that data were missing due to various reasons not under the control of the study teams (e.g., time constraints during visit).

Figure S4. Predictor Matrix Used in Multiple Imputation of Missing Data

*Note*. Variables in the final dataset were assessed as predictors of missingness. Binary indicators of children belonging to subsets of the present sample as well as the Children’s Global Assessment Scale (CGAS) were used as auxiliary variables. Continuous variables were included as predictors if they differentiated between children with and without missing data on a variable by standardized mean differences (Cohen’s *d*) ≥ .20, whereas categorical variables were included if they significantly (*p* < .05) differentiated between children with and without missing data on a variable in logistic regression models (citation). Proportional odds and predictive mean matching imputation methods were used to predict missing ordinal and continuous values, respectively. Forty datasets were imputed. The light blue squares in the figure demonstrate where a predictor on the x-axis was used to impute values for each variable on the y-axis. PFC = Preschool Feelings Checklist-Scale, MCA = My Child 2, ERC LN/ER = Emotion Regulation Checklist Lability/Negativity and Emotion Regulation, CBCL INT/EXT/TOT = Child Behavior Checklist Internalizing/Externalizing/Total Problems, BRIEF GEC = Behavior Rating Inventory of Executive Function Global Executive Composite, BIS/BAS = Behavioral Inhibition/Activation Scales, NOA = Neurodevelopment of Overcontrol and Anxiety Study, and PCIT HC/CC= Parent-Child Interaction Therapy–Emotion Development Study Healthy/Community Control.

Figure S5. Hypothesized Factor Models of the Preschool Feelings Checklist–Scale (PFC-S)

*Note*. Observed PFC-S items and latent factors are depicted in rectangles and circles, respectively. Curved lines with double-headed arrows represent correlations. Straight arrows with single-headed arrows represent factor loadings. Black/dark straight lines depict loadings expected to be primary/cardinal per factor, whereas gray/light straight lines depict loadings expected to cross-load onto 2 factors (citation). Variances, residual terms, and all other loadings in these exploratory structural equation models were not depicted for figure simplicity. Item 16 was removed from the present investigation due to prior work demonstrating its low communality with the rest of the PFC-S items (citation).

^a^ Reverse-coded item.

Table S5. Hypothesized Correlations between PFC-S Factors and Construct Validation Measures

|  |  | PFC-S Factors | | | |
| --- | --- | --- | --- | --- | --- |
| Caregiver-Report Measures | | G | SBA | EBD | EGS |
| Correlation Model 1 | | + | + | + | + |
|  | ERC Lability/Negativity | – | – | – | – |
|  | ERC Emotion Regulation |  |  |  |  |
|  | Behavioral Inhibition Scale (BIS) | + | + | + | + |
|  | Behavioral Activation Scale (BAS) | *ns* | *ns* | + | *ns* |
|  | BAS Reward Responsiveness | *ns* | – | *ns* | *ns* |
|  | BAS Drive | + | + | + | + |
|  | BAS Fun-Seeking | *ns* | *ns* | + | *ns* |
|  | BDI-II Caregiver Depressive Severity | + | + | + | + |
| Correlation Model 2 | |  |  |  |  |
|  | KSADS-EC Anhedonia Severity | *+* | *+* | *ns* | *ns* |
|  | BRIEF Global Executive Composite | + | *ns* | + | *ns* |
|  | MC2 Guilt Feelings | + | *ns* | *ns* | + |

*Note*. Expected positive correlations shown by “+” whereas expected negative correlations shown by “–” (citation). PFC-S = Preschool Feelings Checklist-Scale, G = General Factor, SBA = Social and Behavioral Anhedonia, EBD = Emotional and Behavioral Dysregulation, EGS = Excessive Guilt and Sadness, ERC = Emotion Regulation Checklist, BDI-II = Beck Depression Inventory–II, KSADS-EC = Kiddie Schedule for Affective Disorders and Schizophrenia Early Childhood, BRIEF = Behavior Rating Inventory of Executive Function, MC2 = My Child 2, and *ns* = not significant.

Table S6. Hypothesized Group Distinctions based on PFC-S Factor Score Optimal Cutoffs

| KSADS-EC-Based Diagnostic Groups | | |  | PFC-S Factors | | | |
| --- | --- | --- | --- | --- | --- | --- | --- |
| Reference | Condition | |  | G | SBA | EBD | EGS |
| No Diagnoses | Any Single Diagnosis | |  | + | *ns* | + | *ns* |
| No Diagnoses | Any Co-Occurring Diagnoses | |  | + | + | + | *ns* |
| No Diagnoses | MDD/CO– | |  | + | + | + | + |
| No Diagnoses | MDD/CO+ | |  | + | + | + | + |
| Any/MDD– | MDD/CO– | |  | *ns* | + | *ns* | + |
| Any/MDD– | MDD/CO+ | |  | *ns* | + | *ns* | + |
| ANX/MDD– | MDD/ANX– | |  | *ns* | + | *ns* | *ns* |
| CBCL-Based Groups | | |  |  |  |  |  |
| Dimension | Non-Clinical (*n*) | Clinical (*n*) |  |  |  |  |  |
| Internalizing | 301 | 202 |  | + | + | + | + |
| Externalizing | 333 | 170 |  | + | *ns* | + | – |
| Total | 295 | 208 |  | + | *ns* | + | *ns* |

*Note*. Expected distinctions from logistic regression models are shown by “+” and “–” based on whether the Condition/Clinical group would be expected to show higher and lower PFC-S scores than the Reference/Non-Clinical group, respectively. KSADS-EC = Kiddie Schedule for Affective Disorders and Schizophrenia Early Childhood, PFC-S = Preschool Feelings Checklist-Scale, G = General Factor, SBA = Social and Behavioral Anhedonia, EBD = Emotional and Behavioral Dysregulation, EGS = Excessive Guilt and Sadness, MDD = Major Depressive Disorder, CO– = no co-occurring diagnoses, CO+ = with co-occurring diagnoses, Any = Any Diagnosis, MDD– = no MDD, ANX = 1+ anxiety diagnosis, ANX– = no anxiety diagnoses, and *ns* = not significant.

Table S7. Factor Loadings of the 3-CF and 3-Bifactor Models of the PFC-S

|  |  | 3-CF Model | | |  | 3-Bifactor Model | | | |
| --- | --- | --- | --- | --- | --- | --- | --- | --- | --- |
| Items |  | SBA | EBD | EGS |  | General | SBA | EBD | EGS |
| 1. Interested in playing with other kids ^a^ |  | **.832** | -.028 | .009 |  | **.707** | **.374** | -.186 | -.148 |
| 3. Engages in activities that make him/her happy ^a^ |  | **.735** | .094 | .077 |  | **.693** | **.528** | .101 | .073 |
| 5. Seems less excited about play/activities than other kids |  | **.627** | .036 | .326 |  | **.839** | **.270** | -.102 | .083 |
| 9. Enjoys activities and play ^a^ |  | **.802** | .063 | -.023 |  | **.642** | **.589** | .079 | -.006 |
| 10. Keeps to him/herself |  | **.539** | -.028 | .132 |  | **.577** | **.153** | -.183 | -.060 |
| 17. Seems to be very tired and low energy |  | **.240** | **.621** | .043 |  | **.856** | **-.177** | .084 | -.166 |
| 6. Has difficulty sleeping at night |  | -.022 | **.932** | -.188 |  | **.585** | .036 | **.549** | -.047 |
| 7. Whines/cries |  | -.009 | **.559** | .055 |  | **.494** | .018 | **.340** | .062 |
| 8. Can’t pay attention to games/tasks |  | -.137 | **.724** | .210 |  | **.662** | -.097 | **.429** | .152 |
| 4. Has a lot of trouble following simple directions |  | -.061 | **.843** | -.069 |  | **.559** | .080 | **.572** | .047 |
| 11. Pretend plays about scary/sad things |  | -.038 | **.470** | .265 |  | **.605** | -.120 | **.208** | .126 |
| 23. Seems more irritable/grouchy than other kids his/her age |  | .174 | **.576** | .079 |  | **.772** | -.175 | **.089** | -.086 |
| 14. Is having problems at preschool/daycare |  | .126 | **.665** | .159 |  | **.829** | -.061 | **.268** | .030 |
| 21. Used to behave his/her age but now acts younger |  | **.231** | .159 | **.416** |  | **.691** | .033 | .002 | **.178** |
| 22. Has trouble getting along with other kids |  | .117 | **.348** | **.247** |  | **.602** | .014 | .157 | **.118** |
| 2. Appears sad or says s/he feels sad |  | .175 | .224 | **.455** |  | **.703** | .084 | .114 | **.249** |
| 13. Seems to lack confidence |  | -.216 | -.016 | **1.053** |  | **.632** | -.049 | .100 | **.687** |
| 12. Blames him/herself for things |  | .154 | .047 | **.663** |  | **.724** | .019 | -.027 | **.348** |
| 15. Not excited about things other children his/her age find exciting |  | .549 | .070 | **.274** |  | **.772** | .192 | -.109 | .043 |
| 18. Seems to feel overly guilty |  | -.067 | -.152 | **1.070** |  | **.665** | .013 | -.010 | **.648** |
| 19. Seems lonely |  | .229 | .212 | **.480** |  | **.783** | .040 | .042 | **.222** |
| 20. Has unusually increased or decreased appetite |  | .118 | .461 | **.152** |  | **.621** | .014 | .216 | .063 |

*Note*. Factor loadings are shown per item and factor of each model. Bolded numbers show expected primary/cardinal loadings, regular black numbers show meaningful loadings (*p* < .05) not expected to be primary/cardinal, and gray numbers show non-meaningful (*p* ≥ .05) loadings (citation). Item 16 was removed from the present investigation due to prior work demonstrating its low communality with the rest of the PFC-S items (citation). 3-CF = 3-correlated-factors model, 3-Bifactor = bifactor model with 3 group factors, SBA = Social and Behavioral Anhedonia, EBD = Emotional and Behavioral Dysregulation, and EGS = Excessive Guilt and Sadness.

^a^ Reverse-coded item.

Figure S6. Construct Validation of the PFC-S General Factor Composite Mean Scores

*Note*. Significant (*p* < .05) correlations are depicted by black/dark text and shapes, whereas non-significant (*p* ≥ .05) correlations are depicted by gray/light text and shapes. The dashed line is a visual aid for *r* = 0. Dots depict point estimates and the solid lines depict 95% confidence intervals. While polyserial and point-biserial correlations were respectively estimated for KSADS-EC Anhedonia scores and Sex (0 = female, 1 = male), Pearson’s correlations were estimated for all other measures. PFC-S = Preschool Feelings Checklist-Scale, ERC = Emotion Regulation Checklist, BIS/BAS = Behavioral Inhibition/Activation Scales, BDI-II = Beck Depression Inventory–II, BRIEF GEC = Behavior Rating Inventory of Executive Function Global Executive Composite, MC2 = My Child 2, KSADS-EC = Kiddie Schedule for Affective Disorders and Schizophrenia Early Childhood.
